# Dietary ZnO nanoparticles alters intestinal microbiota and inflammation response in weaned piglets

**DOI:** 10.18632/oncotarget.17612

**Published:** 2017-05-04

**Authors:** Tian Xia, Wenqing Lai, Miaomiao Han, Meng Han, Xi Ma, Liying Zhang

**Affiliations:** ^1^ State Key Laboratory of Animal Nutrition, China Agricultural University, Beijing, China

**Keywords:** nano zinc oxide, antioxidant enzyme, inflammation response, intestinal microbiota, weaning piglet, Immunology and Microbiology Section, Immune response, Immunity

## Abstract

The present study was carried out to determine whether low dose of zinc oxide nanoparticles (Nano-ZnO) could serve as a potential substitute of pharmacological dose of traditional ZnO in weaned piglets. 180 crossbred weaning piglets were randomly assigned to 3 treatments. Experimental animals were fed basal diet supplemented with 0 mg Zn/kg (Control), 600 mg Zn/kg (Nano-ZnO) and 2000 mg Zn/kg (ZnO) for 14 days. On day 14 after weaning, the piglets fed Nano-ZnO did not differ from those fed traditional ZnO in growth performance and jejunal morphology, while Nano-ZnO treatment could significantly alleviate the incidence of diarrhea (*P* < 0.05). In jejunum, the mRNA expressions of intestinal antioxidant enzymes and tight junction proteins were increased (*P* < 0.05) in Nano-ZnO treatment. In ileum, the expression levels of IFN-γ, IL-1β, TNF-α and NF-κB were decreased (*P* < 0.05). Gene sequencing analysis of 16S rRNA revealed that dietary Nano-ZnO increased the bacterial richness and diversity in ileum, while decreased both of them in cecum and colon. Specifically, the relative abundances of *Streptococcus* in ileum, *Lactobacillus* in colon were increased, while the relative abundances of *Lactobacillus* in ileum, *Oscillospira* and *Prevotella* in colon were decreased (*P* < 0.05). In conclusion, our data reveal that low dose of Nano-ZnO (600 mg Zn/kg) can effectively reduce piglet diarrhea incidence, similar to high dose of traditional ZnO (2000 mg Zn/kg), which may be mediated by improving intestinal microbiota and inflammation response in piglets, and help to reduce zinc environmental pollution.

## BACKGROUND

Weaning is commonly associated with oxidative stress, barrier dysfunction and the perturbation of gut microbiota especially in weaned piglets, which is responsible for the growth retardation, villus atrophy and diarrhea [1, 2]. In current animal husbandry industry, pharmacological dose of dietary ZnO (2000-3000 mg Zn/kg) has been widely used due to its efficient function on promoting growth and relieving diarrhea of weaned piglets. Moreover, these functions may be accomplished by improving barrier function, regulating oxidation state, modulating immune response and altering microbiota [3]. However, pigs consume relatively higher level of Zn will excrete more in urine and feces, which raises concerns on environmental pollution [4], and causes negative evaluation of ZnO using in weaned piglets diet.

Nano-ZnO is an inorganic zinc product ranging in particle size from 1 to 100 nm. It has been reported that Nano-ZnO differed from ordinary ZnO due to its unique chemical and physical properties, including the effect of volume, surface, quantum size and macro quantum tunnel [5], in addition, Nano-ZnO only partly dissolved in gastric fluid *in vivo* [6], and showed a limited solubility in neutral environments [7]. Thus, Nano-ZnO is likely absorbed as the intact particulate form and as zinc ions [8]. Studies about Nano-ZnO application mainly focused on the hazards or toxicity in a dose- and time-dependent manner, which was probably caused by reactive oxygen species, oxidant injury or excitation of inflammation [9]. In addition, a wide range of antibacterial activities with the damage of bacterial cell membrane has also been studied [10]. It has been demonstrated that Nano-ZnO moderated the antioxidant activity and revealed an excellent anti-inflammatory property by suppressing the expression of inflammatory cytokines in a dose-dependent manner. Nano-ZnO supplementation has been demonstrated to increase gain weight, feed conversion and improve the antioxidant capacity in broilers [11]. An enhancing immune response of cows was also found [12].

The present study aimed to evaluate whether the low level of Nano-ZnO has a comparable effect of pharmacological dose of ZnO on benefitting piglet health. Our research revealed that the positive function of Nano-ZnO might be accomplished by improving intestinal morphology, regulating the expressions of antioxidant enzymes, tight junction proteins, growth marker genes and inflammatory cytokines, and modulating bacterial community.

## RESULTS

### Dietary Nano-ZnO improved growth performance and jejunal morphology of weaned piglets

Feed:gain of weaning piglets in traditional ZnO treatment was significantly lower than that in the control group (*P* < 0.05), but did not differ from that in the Nano-ZnO treatment (*P* > 0.05). Incidence of diarrhea was alleviated by the addition of Nano-ZnO and traditional ZnO when compared with the control group (*P* < 0.01), while piglets in Nano-ZnO treatment had higher diarrhea incidence than piglets in traditional ZnO group (*P* < 0.05). No significant differences were found in average daily gain and average feed intake among treatments (*P* > 0.05) (Table 1).

**Table 1:** Growth performance and diarrhea incidence in weaning piglets. ^1^

Variable	Control	Nano-ZnO	ZnO	SEM	*P* value
Initial body weight, kg	7.43 ± 0.13	7.47 ± 0.13	7.36 ± 0.15	0.24	0.96
Final body weight, kg	11.6 ± 0.32	12.1 ± 0.24	12.0 ± 0.29	0.50	0.77
Average daily gain, g	330 ± 29.6	341 ± 29.7	344 ± 31.7	16.0	0.34
Average daily feed intake, g	471 ± 35.3	476 ± 33.9	473 ± 36.7	21.3	0.51
Feed:gain	1.44 ± 0.02^a^	1.41 ± 0.02^ab^	1.38 ± 0.02^b^	0.01	0.02
Diarrhea, %	11.0 ± 0.06^a^	6.67 ± 0.45^b^	2.36 ± 0.40^c^	1.02	<0.01

^1^ Values are means ± SEMs; *n* = 36 for initial body weight and final body weight; *n* = 6 for average daily gain, average daily feed intake, feed: gain and diarrhea. Mean values in the same row with different superscript letters differ significantly, *P* < 0.05.

**Figure 1:**
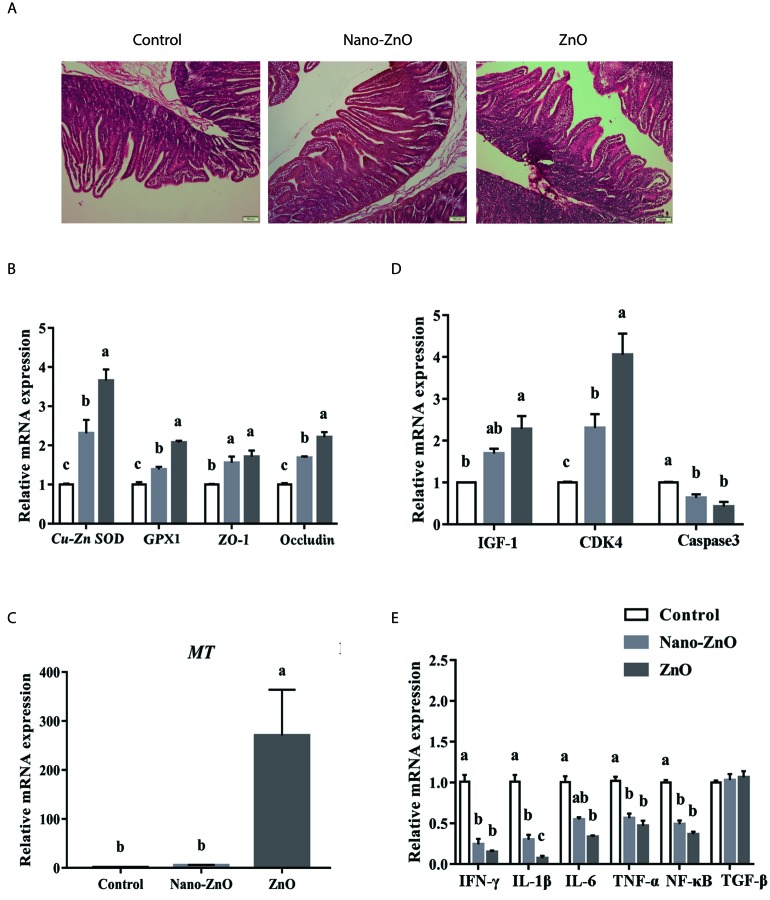
The effects of ZnO treatments on intestinal morphology, and the ralative mRNA expressions of intestinal antioxidant enzymes, tight junction proteins and inflammatory cytokines **A.** The jejunum in different groups were stained by HE. Histology images of jejunal morphology were collected. **B.** The relative mRNA expressions of Cu-Zn SOD, GPX1, ZO-1, and Occludin were detected. As to MT, its relative mRNA expression was also measured in jejunum **C.**. The relative mRNA expressions of IGF-1, CDK4 and Caspase3 in jejunum of weaning piglets were also measured **D.**. In addition, the IFN-γ, IL-1β, IL-6, TNF-α, NF-κB and TGF-β were detected by quantitative RT-PCR **E.**. β-actin was used as an internal standard for normalization. Values are means ± SEMs, *n* = 6. Different superscripts within each group mean significant difference (*P* < 0.05). Control, corn-soybean meal basal diet; Cu-Zn SOD, Cu-Zn superoxide dismutase; GPX1, glutathione peroxidase 1; MT, metallothionein; IGF-1, insulin-like growth factor 1; Nano-ZnO, corn-soybean meal basal diet supplemented with 600 mg Zn/kg as nano zinc oxide; ZnO, corn-soybean meal basal diet supplemented with 2000 mg Zn/kg from zinc oxide.

Jejunal villus height and the ratio of villus height to crypt depth of weaning piglets fed traditional ZnO were significantly higher than piglets in the control group (*P* < 0.05), but showed no significant difference when compared with Nano-ZnO treatment (*P* > 0.05) (Figure 1A). There was no significant difference in crypt depth of jejunum among treatments (*P* > 0.05) (Table 2).

**Table 2:** Jejunal Morphology of weaning piglets.^1^

Variable	Control	Nano-ZnO	ZnO	SEM	*P* value
Villus height, μm	327 ± 7.32^b^	340 ± 6.89^ab^	353 ± 8.78^a^	9.06	0.05
Crypt depth, μm	148 ± 5.36	145 ± 4.73	146 ± 5.18	6.09	0.69
Villus height: crypt depth	2.22 ± 0.07^b^	2.35 ± 0.08^ab^	2.43 ± 0.07^a^	0.04	0.04

^1^ Values are means ± SEMs, *n* = 6. Mean values in the same row with different superscript letters differ significantly, *P* < 0.05.

### Dietary Nano-ZnO activated the expression of Cu-Zn SOD, GPX1, ZO-1, Occludin and MT in the jejunal tissues

Compared with the control group, dietary supplementation with Nano-ZnO and traditional ZnO significantly increased the relative mRNA expressions of Cu-Zn SOD, GPX1, ZO-1 and Occludin in jejunum (*P* < 0.05). Moreover, their expressions in Nano-ZnO treatment were lower than those in traditional ZnO treatment (*P* < 0.05). In addition, the relative MT mRNA expression by 5.4 or 270.4 fold was increased after supplementing Nano-ZnO and traditional ZnO, and interestingly, the relative mRNA expression of MT in the traditional ZnO group was dramatically higher than that in other two groups (*P* < 0.05) (Figure 1B-1C).

### Dietary Nano-ZnO improved the expression of inflammatory cytokines in the jejunal and ileal tissues

Compared with the control, the relative mRNA expression of CDK4 was elevated but the relative mRNA expression of Caspase3 was reduced by Nano-ZnO and traditional ZnO significantly (*P* < 0.01). While the mRNA expression of CDK4 in the Nano-ZnO treatment was significantly lower than that in the traditional ZnO group (*P* < 0.05). The mRNA expression of IGF-1 was increased by traditional ZnO compared with the control group (*P* < 0.05), but no significant differences were found in mRNA expression of IGF-1 between traditional ZnO and Nano-ZnO treatments (*P* > 0.05) (Figure 1D).

Dietary supplementation with Nano-ZnO and traditional ZnO significantly decreased the relative mRNA expression of IFN-γ, IL-1β, TNF-α and NF-κB in comparison to control animals (*P* < 0.01). The mRNA expression of IFN-γ, TNF-α and NF-κB did not differ between Nano-ZnO and traditional ZnO treatments (*P* > 0.05), while as to IL-1β, Nano-ZnO was significantly higher than the traditional ZnO treatment (*P* < 0.01). When detected the expression of IL-6, the results showed that this in the traditional ZnO group was significantly lower than the control group (*P* < 0.01), but did not differ from the Nano-ZnO treatment (*P* > 0.05). No significant difference was found in TGF-β among treatments (*P* > 0.05) (Figure 1E).

### Dietary Nano-ZnO altered the richness and diversity of bacterial community in the ileal, cecal, and colonic contents

**Figure 2:**
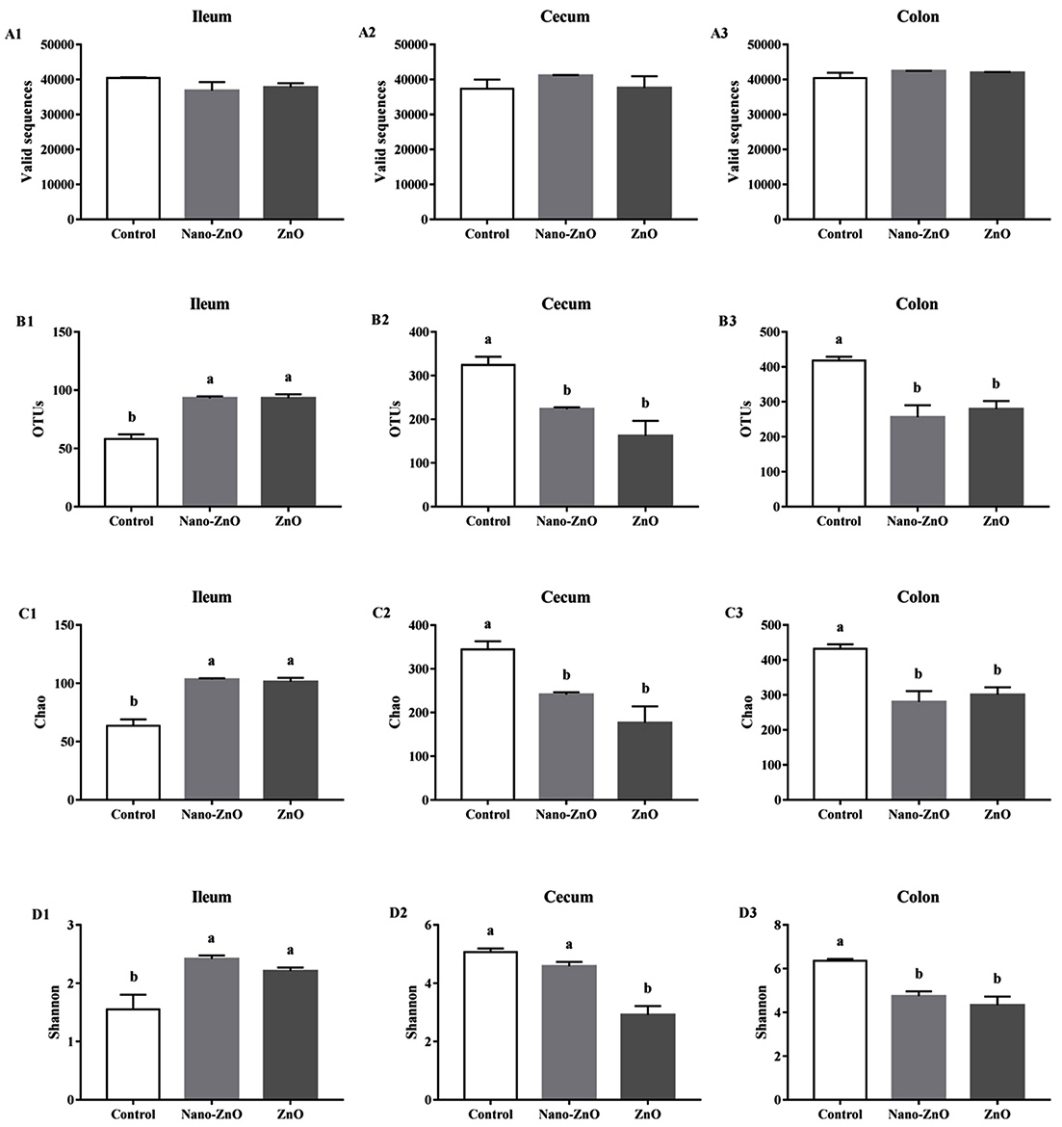
The richness and diversity of bacterial community in the ileal, cecal and colonic contents of weaning piglets Valid sequences **A.** and operational taxonomic units (OTUs) **B.** were clustered from sequences with similarity scores ≥ 0.97 respectively in ileum, cecum, and colon. The microbiota richness was estimated by Chao 1 value and the estimates in ileum, cecum and colon were all gained **C.**. Shannon index could indicate the bacterial diversity, which was measured among ileum, cecum and colon separately **D.** Values are means ± SEMs, *n* = 3. Mean values followed by different letters differ significantly, *P* < 0.05. Control, corn-soybean meal basal diet; Nano-ZnO, corn-soybean meal basal diet supplemented with 600 mg Zn/kg as nano zinc oxide; ZnO, corn-soybean meal basal diet supplemented with 2000 mg Zn/kg from zinc oxide.

After size filtering, quality control and chimera removal, a total of 690231, 696240, and 748887 valid sequences in the ileal, cecal, and colonic contents were obtained respectively. While valid sequences in the ileal, cecal, and colonic digesta showed no significant difference among treatments (*P* > 0.05) (Figure 2A). Operational taxonomic unit (OUT) numbers of bacterial community were classified from valid sequence with 97% similarity. OTUs, Chao 1 value and Shannon index of bacterial community were significantly higher in Nano-ZnO and traditional ZnO treatments (*P* < 0.05) (Figure 2B), but lower in the colonic content (*P* < 0.05) (Figure 2B3-2D3). In addition, the OTUs and Chao 1 value in the cecal content were markedly decreased by Nano-ZnO and traditional ZnO (*P* < 0.01) (Figure 2B2-2C2), and the OTUs and Chao 1 value showed no significant difference between Nano-ZnO and traditional ZnO treatments (*P* > 0.05). However, the Shannon index in the traditional ZnO treatment was significantly lower than other two treatments (*P* < 0.01) (Figure 2D2).

**Figure 3:**
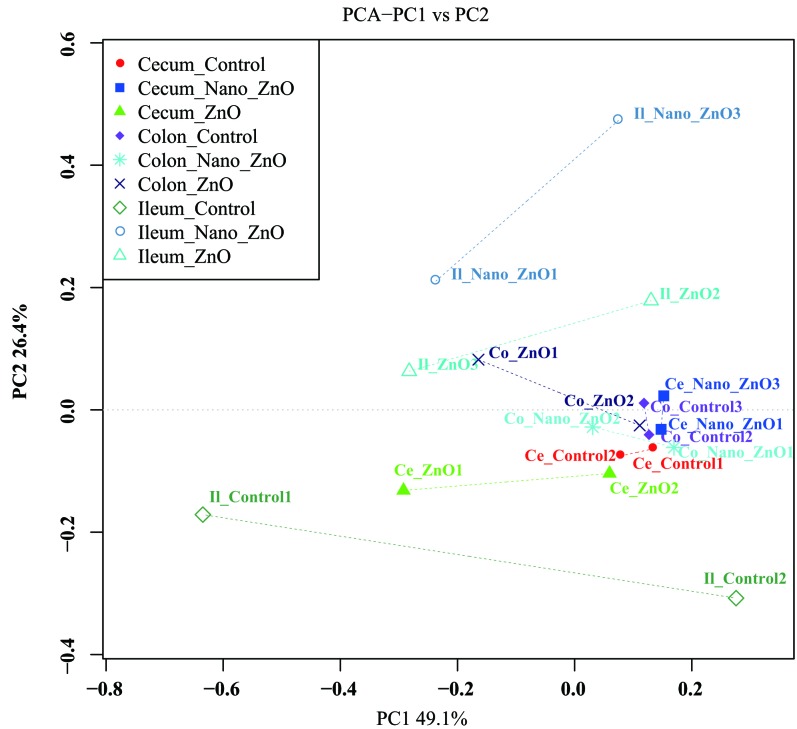
Ileal, cecal and colonic principal coordinate analysis (PCoA) and Venn diagrams of bacterial communities Unweighted PCoA by ileal and colonic bacterial microbiota. Ce-/Co-/Il-control, the cecal/colonic/ileal digesta in the control treatment; Ce-//Co-/Il-Nano-ZnO, the cecal/colonic/ileal digesta in the Nano-ZnO treatment; Ce-/Co-/Il-ZnO, the cecal/colonic/ileal digesta in the ZnO treatment.

The PCA with unweighted UniFrac distances presents discrepancy among samples. The samples clustered according to intestinal segments, and foregut’s (ileal) samples separated with hindgut’s (cecal and colonic) ones obviously (Figure 3), indicating that there were distinct differences in bacterial community between foregut and hindgut.

### Dietary Nano-ZnO improved the baterial community structures in the ileal, cecal, and colonic contents

**Figure 4:**
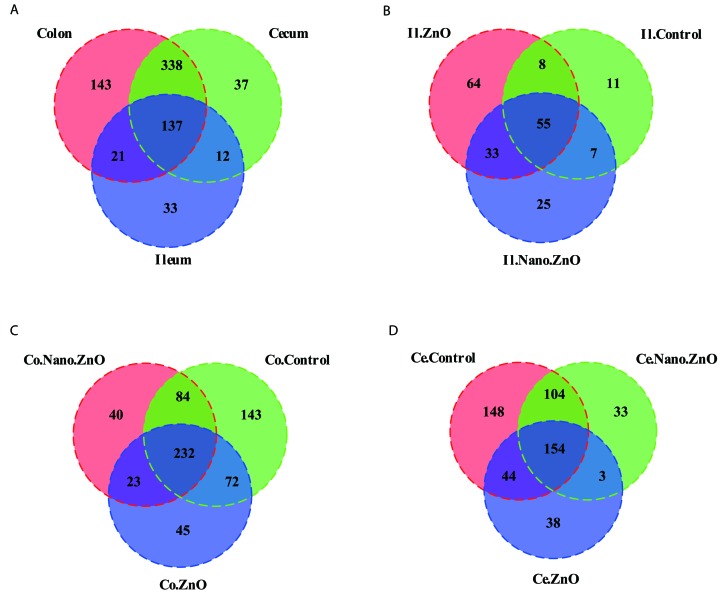
Venn diagrams of bacterial communities and bacterial OTUs in different parts of intestine among three treatments **A.** Venn diagrams illustrating overlap of bacterial OTUs in ileal, cecal and colonic samples. **B.**-**D.** Venn diagrams illustrating overlap of bacterial OTUs in **B.** Ileum, **C.** Cecum, **D.** Colon among three treatments. Ce./Co./Il.Control, the cecal/colonic/ileal digesta in the control treatment; Ce./Co./Il.Nano.ZnO, the cecal/colonic/ileal digesta in the Nano-ZnO treatment; Ce./Co./Il.ZnO, the cecal/colonic/ileal digesta in the ZnO treatment.

16S rRNA profiles for each experimental group in the ileum, colon and cecum were very dissimilar. The OTU composition and abundance were relatively similar between the colon, ileum and cecum content in the same treatment groups (Figure 4A). Meanwhile, Venn diagrams were constructed to represent the shared richness among the ileal, cecal, and colonic digesta samples (Figure 4B-4D). Much more unique OTUs were detected among the ileal, cecal, and colonic digesta samples as well.

**Figure 5:**
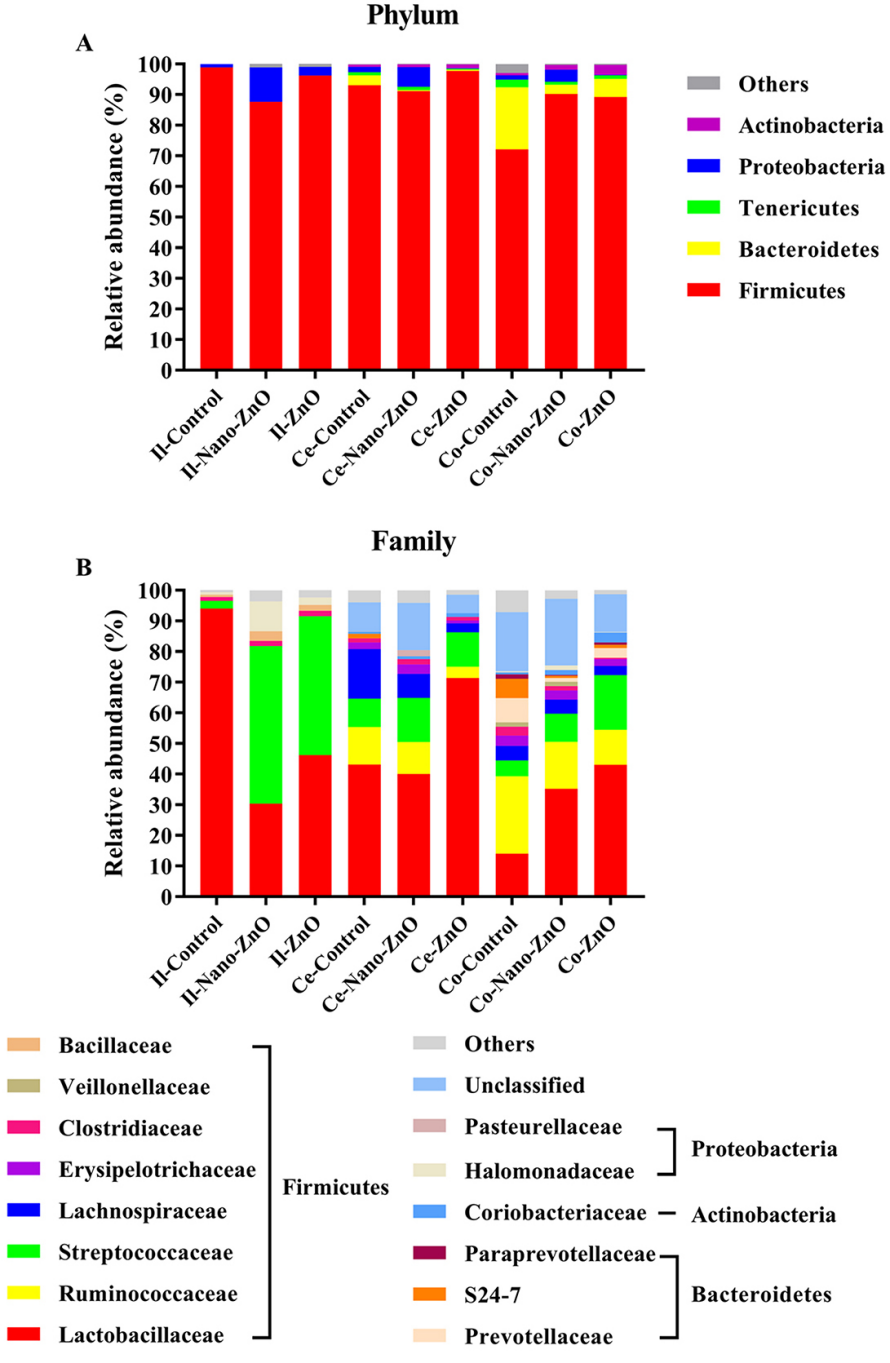
Effects of Nano-ZnO and ZnO on ileal, cecal and colonic bacterial community of weaning pigs Relative read abundance of different bacterial phylum **A.** and families **B.** within different communities in the ileal, cecal and colonic digesta in different treatments was detected. Values were means, *n* = 3. Phyla and families with proportion less than 1% were not listed. Control, corn-soybean meal basal diet; Nano-ZnO, corn-soybean meal basal diet supplemented with 600 mg Zn/kg as nano zinc oxide; ZnO, corn-soybean meal basal diet supplemented with 2000 mg Zn/kg from zinc oxide. Ce-/Co-/Il-control, the cecal/colonic/ileal digesta in the control treatment; Ce-//Co-/Il-Nano-ZnO, the cecal/colonic/ileal digesta in the Nano-ZnO treatment; Ce-/Co-/Il-ZnO, the cecal/colonic/ileal digesta in the ZnO treatment.

The ileal microbiota in weaning piglets mainly consisted of the Firmicutes and Proteobacteria at phylum level. The supplementation of Nano-ZnO caused notable shifts in relative abundance, consisting of a decrease in Firmucutes and an increase in Proteobacteria after 14 d post-weaning (*P* < 0.05) (Figure 5A).

At family level, Firmicutes were mainly composed of Lactobacillaceae, Streptococcaceae, Clostridiaceae, and Bacillaceae, while Halomonadaceae was the primary family of Proteobacteria (Figure 5B). Lactobacillaceae was the richest family in the control group, but its proportion dropped dramatically from 94.08% to 30.37% and 46.17% after adding Nano-ZnO and traditional ZnO (*P* < 0.01). On the contrary, the relative abundances of Streptococcaceae and Bacillaceae significantly increased, from 2.47% to 51.44% and 45.36% and from 0.74% to 3.12% and 1.88%, respectively (*P* < 0.01). In addition, the increased abundance of Proteobacteria in the Nano-ZnO treatment was mainly due to a significant increase of Halomonadaceae.

At genus level, *Lactobacillus, Streptococcus, Bacillus* and *Halomonas* were predominant genera of Lactobacillaceae, Streptococcaceae, Bacillaceae and Halomonadaceae, respectively, and the changed tendencies of these genera were consistent with the tendencies of responding families as Nano-ZnO and traditional ZnO were added to the basal diet.

In the cecal contents, Firmicutes, Bacteroidetes, Proteobacteria, Tenericutes, and Actinobacteria were five major phyla of microbiota (Figure 5A). The relative abundance of Firmicutes increased from 93.05% to 97.72% when traditional ZnO was added to the basal diet (*P* < 0.05). At family level, Firmicutes were mainly composed of Lactobacillaceae, Streptococcaceae, Lachnospiraceae, Ruminococcaceae, Erysipelotrichaceae, and Clostridiaceae (Figure 5B). In addition, S24-7, Pasteurellaceae, and Coriobacteriaceae were the primary families of Bacteroidetes, Proteobacteria and Actinobacteria, respectively. Comparing with other two treatments, the proportion of Lactobacillaceae was significantly increased by traditional ZnO treatment (*P* < 0.05), while the relative abundances of Lachnospiraceae, Ruminococcaceae and Erysipelotrichaceae were markedly decreased (*P* < 0.05). No significant differences were found in those above-mentioned bacteria between the control and Nano-ZnO treatments (*P* > 0.05). However, we still observed that the unidentified bacteria in the Nano-ZnO treatment were significantly higher than other two groups (*P* < 0.05). Down to the genus level, *Lactobacillus* occupied an important position in Lactobacillaceae, and *Streptococcus* in Streptococcaceae, and their proportions varied consistently with their affiliated families. Lachnospiraceae primarily consisted of *Blautia, Coprococcus, Dorea, Lachnospira,* and *Roseburia*. Among them, the relative abundances of *Dorea* and *Roseburia* tended to show different decreases after treatment with Nano-ZnO and/or traditional ZnO in the basal diet. *Butyricicoccus, Faecalibacterium, Oscillopira* and *Ruminococcus* are the main genus of Ruminococcaceae, and the proportion of *Ruminococcus* was significantly decreased in Nano-ZnO and traditional ZnO treatments when compared with the control group (*P* < 0.05). However, there were still about 10.22%-27.94% cecal bacteria that were unidentified at genus level.

In the colonic contents, Firmicutes, Bacteroidetes, Actinobacteria, Proteobacteria and Tenericutes were dominant phyla (Figure 5A). When treatingg with Nano-ZnO and traditional ZnO, the relative abundance of Firmicutes was significantly increased (*P* < 0.01), while the abundance of Bacteroidetes was decreased (*P* < 0.01). At family level, Firmicutes was mainly consisted of Lactobacillaceae, Streptococcaceae, Clostridiaceae, Lachnospiraceae, Ruminococcaceae, Veillonellaceae and Erysipelotrichaceae (Figure 5B). Bacteroidetes was mainly composed of Prevotellaceae, S24-7 and Paraprevotellaceae, and the Coriobacteriaceae and Halomonadaceae were the primary families of the Actinobacteria and Proteobacteria. Compared with the control group, the relative abundance of Lactobacillaceae in both two treatments was increased significantly (*P* < 0.05), while the relative abundances of Prevotellaceae and S24-7 were markedly decreased (*P* < 0.05). In addition, by treating with traditional ZnO, the proportion of Lachnospiraceae was significantly decreased compared to the control group (*P* < 0.05). Down to the genus level, *Collinsella, Prevotella, Lactobacillus, Streptococcus, SMB53, Oscillospira, Bulleidia* and *Halomonas* were predominant genera of Coriobacteriaceae, Prevotellaceae, Lactobacillaceae, Streptococcaceae, Clostridiaceae, Ruminococcaceae, Erysipelotrichaceae and Halomonadaceae, respectively. Piglets fed with Nano-ZnO and traditional ZnO diets had a higher level of *Lactobacillus* (*P* < 0.05) and lower *Prevotella* and *Oscillospira* (*P* < 0.05) than piglets fed with the basal diet. Compared with the control group, the relative abundance of *Bulleidia* was significantly reduced by adding traditional ZnO (*P* < 0.05). However, the percent between 22.01%-41.43% of colonic bacterial community was unidentified at genus level.

## DISCUSSION

Dietary pharmacological dose of traditional ZnO (2000-3000 mg Zn/kg) can promote growth and alleviate diarrhea, increase intestinal barrier function and alter intestinal microbiota composition in weaning piglets [13]. However, whether Nano-ZnO can improve piglet health as an alternative to pharmacological dose of traditional ZnO is still unknown. Herein, feed conversion efficiency in the traditional ZnO treatment was significantly increased when compared with the control group, which demonstrated that the pharmacological ZnO supplementation increased the feed efficiency during the post-weaning d 0-10 or d 0-28 [14]. No significant differences in feed conversion efficiency between Nano-ZnO group and traditional ZnO group were found, which indicated that Nano-ZnO with a dose of 600 mg Zn/kg might have a similar effect to traditional ZnO in the concentration of 2000 mg Zn/kg on improving feed efficiency in weaning piglets during post-weaning d 0-14. Incidence of diarrhea was reduced by treating with Nano-ZnO and traditional ZnO, while piglets fed Nano-ZnO had a higher diarrhea incidence than piglets fed with traditional ZnO in this study. This result indicated that a low level of Nano-ZnO inclusion in diet might have a potential function in alleviating post-weaning diarrhea of weaning piglets [3]. Weaning usually changes the architecture and function of piglets gut [15], resulting in a temporary decrease in digestive and absorptive function of the small intestine. Dietary supplementation with pharmacological dose of traditional ZnO has been demonstrated to improve the intestinal morphology of weaning piglets [16]. Similar results were obtained in this study that the villus height and the ratio of villus height to crypt depth of jejunum in traditional ZnO treatment were significantly higher than those in the control group, but did not differ from those in the Nano-ZnO treatment. Therefore, we concluded that the moderate Nano-ZnO might have comparable effects on improving jejunal morphology to high dose of traditional ZnO.

To reveal the potential mechanism, the intestinal barrier function was determined. The mRNA expressions of Cu-Zn SOD, GPX1, ZO-1 and Occludin in the jejunal tissue were elevated by Nano-ZnO and traditional ZnO supplementation. However, between Nano-ZnO and traditional ZnO, the expressions of those above genes were lower in the Nano-ZnO treatment. ZO-1 and Occludin, as key proteins of tight junction, the levels of which expression are consistently associated with the gut barrier function [17]. Our data indicated that dietary supplementation with 600 mg Zn/kg as Nano-ZnO may be rewarding, while the concentration of 600 mg Zn/kg may have a weaker effect than pharmacological dose of traditional ZnO in restoring the decreased antioxidant capacity and injured barrier function in the jejunum tissue caused by weaning stress. More interestingly, traditional ZnO, but not Nnao-ZnO, could dramatically increase the mRNA expression of MT, which is consistent with the report that intestinal mucosa cell from pigs fed 2425 or 3000 mg Zn/kg from ZnO for 14 or 28 d has the greatest concentration or mRNA expression of MT [18]. MT, associated with zinc concentration, not only serves as an antioxidant participating in protective stress responses [19], but also serves as a zinc transporter acting in zinc trafficking and zinc donation to apoproteins [20]. Supplemental traditional ZnO would increase the relative mRNA expression of MT by 270.4 fold, while adding Nano-ZnO would only by 5.4 fold, which further illustrated that ZnO nanoparticle was likely absorbed as both the zinc ions and as the intact particulate form by transcytosis [5, 13], and that ZnO nanoparticle could only partly dissolve in gastric fluid *in vivo* by aggregating together [13]. Gastrointestinal development of weaning piglets relays on the proliferation and differentiation of enterocytes, while the key factors regulating the intestinal development are the expressions of growth marker genes. IGF-1, which can be locally synthesized in the gastrointestinal tract, is an important mediator of the proliferation and differentiation of enterocytes [21]. CDK4 are used as markers to monitor proliferation and apoptosis in the programmed cell death due to its functions in G1 phase of cell cycle that CDK4 could act as a key enzyme [22]. In the current study, the relative mRNA expression of CDK4 in the jejunal tissue was significantly increased in Nano-ZnO and traditional ZnO treatments, however the mRNA expression of Caspase3 was markedly decreased. The results may firstly indicate that Nano-ZnO and traditional ZnO are able to promote proliferation and inhibit apoptosis of enterocyte in the jejunum of weaning piglets. Therefore, dietary supplementation with Nano-ZnO (600 mg Zn/kg) may promote enterocyte growth and inhibit enterocyte apoptosis.

In addition, the mRNA expressions of IFN-γ, IL-1β, TNF-α and NF-κB in the ileal tissue significantly decreased after supplementing Nano-ZnO and traditional ZnO into the basal diet, which was similar to previous study [23]. It has been reported that traditional ZnO or Nano-ZnO supplementation both significantly reduced the expression of NF-κB target gene in the porcine epithelial J2 (IPEC J2) cells or RAW 264.7 Macrophages [24]. The down-regulation of pro-inflammatory cytokines in the present of Nano-ZnO indicated that the weaning-induced inflammation was alleviated by Nano-ZnO at a sub- or non-toxic concentration. Furthermore, Nano-ZnO with the dose of 600 mg Zn/kg might had a potential function that can parallel the pharmacological dose of traditional ZnO in regulating immune and inflammatory responses of weaning piglets [25].

In this study, gene sequencing analysis of 16S rRNA disclosed variations of bacterial community in the ileal, cecal and colonic digesta. In ileum, the bacterial richness was estimated by Chao1 value and diversity was measured by Shannon index, both of which were significantly increased by dietary Nano-ZnO and traditional ZnO treatments. This result was in agreement with previous studies that richness and diversity of microbes in ileum were both elevated after adding traditional ZnO with 2500 mg Zn/kg or 3042 mg Zn/kg [26-27]. In the control treatment, Firmicutes, Lactobacillaceae and *Lactobacillus* dominated bacterial community at phylum, family and genus level, respectively, and their relative abundances were significantly decreased by supplementing Nano-ZnO and/or traditional ZnO. However, in treatments with Nano-ZnO and traditional ZnO, the abundances of Streptococcaceae, Bacillaceae, *Streptococcus* and *Bacillus* were observed to be markedly increased. The results are consistent with reports that Firmicutes and lactic acid bacteria dominate the ileal microbiota, and traditional ZnO would reduce the *Lactobacillus spp*. [26, 28]. The theory that *Streptococcus spp.* seemed to quantitatively replace *L. reuteri* as a major player in the ileum was confirmed in our study to some extent for the increasing level of *Streptococcus* [26]. *Bacillus* can excellently adapt zinc environment as a consequence of spore persistence and its capacity to response to zinc concentration, which may be beneficial to piglet health [29]. The relative abundances of Proteobacteria, Halomonadaceae and *Halomonas* in the Nano-ZnO treatment were significantly higher comparing with the control group, which might mainly due to the increased proportion of *Halomonas* with its strong adaptation to environment rich in zinc [30] and the decreased competition caused by the decreased abundance of *Lactobacillus*. Furthermore, it has been reported that Nano-ZnO had a wide range of antibacterial activities [12] that may involve in both the zinc toxicity from dissolved Zn (II) ions [31] and the production of reactive oxygen species caused by undissolved Nano-ZnO in gastrointestinal tract [13, 27]. However, the antibacterial effect of Nano-ZnO depends on dose, size, shape and other factors [31]. Therefore, both Nano-ZnO and traditional ZnO in this study can enrich the bacterial richness and diversity, which may be beneficial to the construction of a more stable intestinal micro-ecosystem [32].

In cecum, the bacterial richness was decreased by treating with Nano-ZnO and traditional ZnO, while the bacterial diversity was only reduced in traditional ZnO group, which was in agreement with a previous study [22]. Moreover, it also has been investigated that the antibacterial effect of Nano-ZnO is dosage- and time-dependent [33]. Traditional ZnO supplementation (2000 mg Zn/kg) in our study significantly increased the relative abundances of Firmicues, Lactobacillaceae and *Lactobacillus*, and decreased Lachnospiraceae, Ruminococcaceae, Erysipelotrichaceae and *Ruminococcus*. However, feeding with traditional ZnO (2500 mg Zn/kg or 2425 mg Zn/kg) showed a decline of lactic acid bacteria in cecum [22], while no obvious changes in the abundances of the Firmicutes and *Lactobacillus* was observed in the cecal digesta [13]. Researchers reported that all Lactobacillaceae strains possessed the excellently high zinc resistance *in vitro* except *L*.*amylovorus* [32]. The decreased relative abundances of Lachnospiraceae, Ruminococcaceae, Erysipelotrichaceae and *Ruminococcus* might be beneficial to promote growth or alleviate incidence of diarrhea of weaning piglets [34]. Compared to the control group, Nano-ZnO treatment significantly decreased the *Ruminococcus* level, while compared with the traditional ZnO treatment, the unclassified bacterial abundance was increased. It is reported that only 14% of Nano-ZnO could dissolve in gastric fluids [13] and Nano-ZnO had limited solubility in neutral environment [14]. In addition, Nano-ZnO particles are unstable in suspension of water, where they aggregate together and continue to increase size beyond the nanoscale [34]. So the limited impact of Nano-ZnO on cecal microbiota showed in our study might result from the low concentrations of Nano-ZnO and zinc ions dissolved from Nano-ZnO.

In colon, the richness and diversity of bacteria were significantly decreased in Nano-ZnO and traditional ZnO treatments, which was also consistent with a previous study [14]. Moreover, the Nano-ZnO particles have a wide range of antibacterial activities toward various microorganisms [35]. Dietary supplementation with Nano-ZnO and traditional ZnO significantly decreased the relative abundances of Bacteroidetes, Prevotellaceae, S24-7, *Prevotella* and *Oscillospira* and increased the relative abundances of Firmicutes, Lactobacillaceae and *Lactobacillus*. However, high concentration of dietary traditional ZnO (2500 mg Zn/kg or 2425 mg Zn/kg) significantly decreased the abundance of lactic acid bacteria, such as *Lactobacillus spp*. in colon [22]. Adding traditional ZnO in the concentration of 3.1 g/kg was difficult to apparently change the abundances of the Bacteroidetes, Firmicutes and *Lactobacillus* [36]. Liedtke and Vahjen have reported that 10 of 11 *Lactobacillaceae* strains showed high zinc resistance. So the different results in different studies may result from dietary zinc contents, resistances of bacteria and so on. The relative abundances of Lachnospiraceae and *Bulledia* in the traditional ZnO treatment were significantly lower than those in the control treatment, which might contribute to reduce the diarrhea incidence [37]. The rival effect of the lower concentration of Nano-ZnO to pharmacological dose of traditional ZnO on colonic bacterial compositions may due to the relative-high concentrated zinc concentration in colon content [38].

In conclusion, dietary supplementation with Nano-ZnO in the concentration of 600 mg Zn/kg was effective in alleviating diarrhea, improving mRNA expressions of antioxidant enzymes, tight junction proteins and growth marker genes in jejunum, inhibiting mRNA expression of inflammatory cytokines in ileum, and regulating ileal and colonic microbiota richness, diversity and stability. Nano-ZnO (600 mg Zn/kg) may be an effective alternative to pharmacological dose traditional ZnO (2000 mg Zn/kg) in improving piglet intestinal health.

## MATERIALS AND METHODS

All procedures used in this experiment were approved by the Animal Care and Use Committee of China Agricultural University (Beijing, China). The ZnO content of Nano-ZnO used in this study was 98.6%, average particle size measured by transmission electron microscopy was 23.0 nm, crystallite size assayed by X-ray diffraction was 25.0 nm, and the bet surface area tested was 48.9 m^2^/g.

### Animals, diets, and experimental protocol

A total of 108 crossbred piglets (Duroc × Landrace × Yorkshire) weaned at 27 ± 1 d of age, with an average initial body weight of 7.42 ± 0.80 kg, were blocked by weight, sex and litter and were assigned to 1 of 3 dietary treatments, with 6 replicates (pens) of 6 piglets (3 males and 3 females). Feed (mash form) and water (nipple drinker) were available *ad libitum* throughout the 14-d feeding trial. The dietary treatments were corn-soybean meal basal diet (Control), corn-soybean meal basal diet supplemented with 600 mg Zn/kg as Nano-ZnO (Nano-ZnO) or 2000 mg Zn/kg from ZnO (ZnO) at the expense of corn. The basal diet was formulated to meet or exceed the nutrient requirements of 7-11 kg pigs recommended by the NRC (2012). The ingredients and chemical compositions of the basal diet are given in Table 3.

**Table 3:** Ingredients and chemical compositions of the basal diet. ^1,2^ (g/kg as fed)

Ingredient composition		Chemical analysis	
Corn	597	Digestible energy^2^ (MJ/kg)	14.9
Soybean meal	90	Dry matter	873
Soy protein concentrate	110	Crude protein	204
Fish meal	47	Calcium	8.2
Whey powder	100	Total phosphorus	6.7
Soybean oil	10	Zn^3^ (mg/kg)	112
Sodium chloride	3	Lysine	16.0
Dicalcium phosphate	8.2	Methionine	5.5
Limestone	6.8	Methionine + Cysteine	8.7
L-Lysine·HCl (78%)	5.5	Threonine	9.7
DL-Methionine (98.5%)	2.7	Tryptophan	2.6
L-Threonine	2.1	Valine	10.1
L-Tryptophan	0.6		
L-Valine	1.0		
Choline chloride	2.0		
Sodium bicarbonate	8.7		
Vitamin-mineral premix^1^	5.0		

^1^ Provided the following per kg of diet : 12,000 IU Vitamin A, 3,000 IU Vitamin D_3_, 30 IU Vitamin E, 2.5 mg Vitamin K_3_, 2.5 mg Thiamine, 4.0 mg Riboflavin, 3.0 mg Pyridoxine, 0.02 mg Vitamin B_12_, 40 mg nicotinic acid, 12.5 mg pantothenic acid, 0.7 mg folic acid, 0.07 mg biotin, 100 mg Fe (FeSO_4_·H_2_O), 150 mg Cu (CuSO_4_), 90 mg Zn (ZnSO_4_), 55 mg Mn (MnSO_4_), 0.25 mg I (KI), 0.30 mg Se (Na_2_SeO_3_).

^2^ All values were obtained from triplicate except digestible energy. Analyzed concentration of zinc in the basal diet. The experimental diets contained zinc at 701 and 2079 mg/kg, respectively.

Piglets and feed were weighed individually at the beginning and the end of the experiment. The average daily gain (ADG), average daily feed intake (ADFI), and feed:gain (F:G) ratio were then calculated. Fecal consistency within each pen was visually assessed at 8:30 each day during this experiment according to the method described by Hill et al. [14]. The scoring system for consistency was as follows: 1 = very firm, 2 = medium firm, 3 = moderately loose, 4 = very loose, and 5 = thin and watery. The occurrence of diarrhea was defined when the feces was thin and watery.

### Chemical analysis of the diets

Diets were sampled at mixing and ground to pass through a 0.15-mm sieve. Dry matter (AOAC procedure 934.01), crude protein (AOAC procedure 990.03), calcium and total phosphorus (AOAC procedure 985.01), and amino acids (AOAC procedure 151 982.30) contents of the diets were analyzed according to the procedures of the AOAC International (2005). Zinc in diets was analyzed by Flame Atomic Absorption Spectrophotometry (AA-6300, Shimadzu Corp., Tyoto, Japan) after samples were wet-digested by using nitric-perchloric acid and diluted with deionized distilled water. The analyzed zinc contents in the control, Nano-ZnO and ZnO treatment diets were 112, 701 and 2079 mg/kg, respectively.

### Sample collection

At the end of the 14-day experiment, one piglet in each pen with body weight and diarrhea incidence closest to the pen average was chosen and fasted overnight (12 h). The abdomen was aseptically opened and the jejunum, ileum, cecum and colon were sampled. The digesta of ileum, cecum and colon were collected aseptically and immediately immersed in liquid nitrogen and stored at -80°C for analysis of bacterial community.

### Intestinal histomorphometry, RNA isolation and quantitative RT-PCR analysis

Morphological indices were determined using a Leica Image Processing and Analysis System (Leica Imaging Systems Limited, Berlin, Germany). Total RNA isolation and quantitative real-time PCR analysis was conducted as described by Shen et al. [3] with modifications. Briefly, total RNA of jejunum and ileum was extracted using the TRIzol reagent (Invitrogen) after tissue homogenization and treated with DNase I (Invitrogen Life Technologies). The integrity of the extracted RNA was checked by 1% agarose gel electrophoresis and visualisation of intact 18S and 28S ribosomal RNA bands under UV light. Nucleic acid concentrations were measured in triplicate with a UV/VIS-photometer at 260 nm (BioPhotometer, Eppendorf, Germany). Furthermore, purity of the total RNA extracted was determined as the E260/E280 ratio with expected values between 1.8 and 2.0. Reverse transcription was performed using the PrimeScript TM RT Reagent Kit (Takara) with a 1 μg RNA sample. Expression levels of β-actin, Cu-Zn superoxide dismutase (Cu-Zn SOD), glutathione peroxidase 1 (GPX1), metallothionein (MT), zonula occludens protein-1 (ZO-1), occludin, Caspase3, insulin-like growth factor 1 (IGF-1), and cyclin-dependent kinase 4 (CDK4) in jejuna tissue, β-actin, interferon gamma (IFN-γ), interleukin-1 beta (IL-1β), interleukin-6 (IL-6), tumor necrosis factor alpha (TNF-α), nuclear factor kappa-light-chain-enhancer of activated B cells (NF-κB), and transforming growth factor beta (TGF-β) in ileal tissue were analyzed by real-time quantitative PCR with SYBR Premix Ex TaqTM II (Takara), which was performed on complementary DNA samples in ninety-six-well optical plates on a CFX96TM Real-Time System (Bio-Rad). All of the primer sequences for the selected genes were showed in Table 4. The PCR system consisted of 5 μL of 2 × SYBR^®^ premix Ex TaqTM II, 0.3 μL of each forward and reverse primer (10 μmol/L), 4 μL of complementary DNA template, and 0.4 μL of double distilled water. The protocols for all genes included a denaturation program (1 min at 95 °C), amplification and quantification program repeated for 35 cycles (5 s at 95°C, 30 s at 52-63°C, Table 4), followed by the melting curve program at 60-95 °C with a heating rate of 0.1 °C per second and continuous fluorescence measurement.

**Table 4:** Primers used for quantitative RT-PCR. ^1,2^

Target	Primers sequences, 5’-3’		Size (bp)	AT^2^
	Forward	Reverse		
Cu-Zn SOD	CAGGTCCTCACTTCAATCC	CCAAACGACTTCCASCAT	255	60
GPX1	TGGGGAGATCCTGAATTG	GATAAACTTGGGGTCGGT	183	56
MT	GTGAATCCGCGTTGCTCTCTGCT	CTGTGGGGCAGGAGCAGTTGG	138	60
ZO-1	GAGTTTGATAGTGGCGTT	GTGGGAGGATGCTGTTGT	298	52
Occludin	ATCAACAAAGGCAACTCT	GCAGCAGCCATGTACTCT	157	56
Caspase3	TGTGTGCTTCTAAGCCATGG	AGTTCTGTGCCTCGGCAG	158	58
IGF-1	TGCTTCCGGAGCTGTGATCT	CCGACTTGGCAGGCTTGA	67	58
NFκB	AGCTTGCCGTGTCTGCTGCT	CCGCCAAGGAGATGTTGTCG	118	60
CDK4	AGACCTGAAGCCAGAGAACATTC	AAGATACAGCCAACGCTCCAC	195	60
IFN-γ	GAGCCAAATTGTCTCCTTCTAC	CGAAGTCATTCAGTTTCCCAG	140	56
TGF-β	GGACCTTATCCTGAATGCCTT	TAGGTTACCACTGAGCCACAAT	133	60
IL-1β	TGAAGTGCCGCACCCAAAACCT	CGGCTCCTCCTTTGCCACAATCA	175	62
IL-6	CCTGTCCACTGGGCACATAAC	CAAGAAACAACCTGGCTCTGAAAC	253	63
TNF-α	CATCGCCGTCTCCTACCA	CCCAGATTCAGCAAAGTCCA	199	56
β-actin	CTGGAACGGTGAAGGTGA	TTTGGAAAGGCAGGGACT	170	60

^1^ Abbreviations: CDK4, cyclin-dependent kinase 4; Cu-Zn SOD, Cu-Zn superoxide dismutase; GPX1, glutathione peroxidase 1; IGF-1, insulin-like growth factor 1; MT, metallothionein; ZO-1, zonula occludens protein-1

^2^ AT: Annealing temperature in °C.

### Bacterial community

The DNA extraction, PCR amplification, illumina MiSeq sequencing, and bacterial data processing were conducted according to the procedures of Han et al. [31]. DNA extraction of ileal, cecal, and colonic contents was performed according to the instructions of a DNA Stool Mini Kit (Qiagen, Hilden, Germany). The bacterial universal V3-V4 region of the 16S RNA gene was amplified according to PCR bar-coded primers 338F (5’-ACTCCTACGGGAGGCAGCA-3’) and 806R (5’-GGACTACHVGGGTWTCTAAT-3’).

### Statistical analysis

Results are presented as means ± SEMs. All data were analyzed by one-way ANOVA using the general linear model procedures of SAS (SAS Institute Inc., Cary, NC, USA). Difference among treatment means was determined by Student-Newman-Keuls test. *P* values less than 0.05 were considered significant.

## Abbreviations

ADFI: average daily feed intake
ADG: average daily gain
CDK4: cyclin-dependent kinase 4
Cu-Zn SOD: Cu-Zn superoxide dismutase
F:G: feed:gain
GPX1: glutathione peroxidase 1
IGF-1: insulin-like growth factor 1
MT: metallothionein
Nano-ZnO: nano-zinc oxide
OTUs: operational taxonomic units
ZnO: zinc oxide
ZO-1: zonula occludens protein-1
